# An Argument for Amphetamine-Induced Hallucinations in an Invertebrate

**DOI:** 10.3389/fphys.2018.00730

**Published:** 2018-06-25

**Authors:** Anne H. Lee, Cindy L. Brandon, Jean Wang, William N. Frost

**Affiliations:** Department of Cell Biology and Anatomy, The Chicago Medical School, Rosalind Franklin University of Medicine and Science, North Chicago, IL, United States

**Keywords:** hallucinations, invertebrate, *Tritonia*, amphetamine, mollusk

## Abstract

Hallucinations – compelling perceptions of stimuli that aren’t really there – occur in many psychiatric and neurological disorders, and are triggered by certain drugs of abuse. Despite their clinical importance, the neuronal mechanisms giving rise to hallucinations are poorly understood, in large part due to the absence of animal models in which they can be induced, confirmed to be endogenously generated, and objectively analyzed. In humans, amphetamine (AMPH) and related psychostimulants taken in large or repeated doses can induce hallucinations. Here we present evidence for such phenomena in the marine mollusk *Tritonia diomedea*. Animals injected with AMPH were found to sporadically launch spontaneous escape swims in the absence of eliciting stimuli. Deafferented isolated brains exposed to AMPH, where real stimuli could play no role, generated sporadic, spontaneous swim motor programs. A neurophysiological search of the swim network traced the origin of these drug-induced spontaneous motor programs to spontaneous bursts of firing in the S-cells, the CNS afferent neurons that normally inform the animal of skin contact with its predators and trigger the animal’s escape swim. Further investigation identified AMPH-induced enhanced excitability and plateau potential properties in the S-cells. Taken together, these observations support an argument that *Tritonia*’s spontaneous AMPH-induced swims are triggered by false perceptions of predator contact – i.e., hallucinations—and illuminate potential cellular mechanisms for such phenomena.

## Introduction

Invertebrate models have become increasingly valuable for investigating how addictive drugs exert their effects on the nervous system and behavior (Kusayama and Watanabe, 2000; Wolf and Heberlein, 2003; Carvelli et al., 2010; Kennedy et al., 2010; van Swinderen and Brembs, 2010; Alcaro et al., 2011; Huber et al., 2011; Kaun et al., 2012; Musselman et al., 2012; Ramoz et al., 2012; Walters et al., 2012)^1^. The replication of many human drug-related behaviors in invertebrates suggests that underlying mechanisms may have been preserved across diverse nervous systems. For example, the *Drosophila* mutant *Radish* displays reduced attention-like behavior that is partly reversed by the ADHD drug methylphenidate (van Swinderen and Brembs, 2010). In addition, methamphetamine-induced anorexia, and d-amphetamine-, cocaine-, and opioid-associated drug seeking and addiction behaviors have been described in crayfish (Alcaro et al., 2011; Huber et al., 2011), *Drosophila* (Kaun et al., 2012; Walters et al., 2012), *Caenorhabditis elegans* (Carvelli et al., 2010; Musselman et al., 2012), planaria (Kusayama and Watanabe, 2000), and *Lymnaea stagnalis* (Kennedy et al., 2010). Although the behavioral effects of psychostimulants and classical hallucinogens have been studied in invertebrates (Witt, 1971; Nichols et al., 2002; Wolf and Heberlein, 2003), to our knowledge hallucinations themselves have yet to be demonstrated, or even suggested to occur.

Hallucinations are defined as perceptions of stimuli (visual, auditory, tactile) that don’t actually exist (Esquirol, 1965; DSM-IV, 2000). They occur in several psychiatric and neurological diseases, as well as in response to certain drugs of abuse (Asaad and Shapiro, 1986; Brasic, 1998). One of these is the psychostimulant amphetamine (AMPH) and its derivatives. Chronic, or in some cases even single high doses of AMPH can induce a paranoid psychotic state closely resembling that of schizophrenia, complete with vivid hallucinations (Connell, 1958; Angrist and Gershon, 1970; Bell, 1973; Snyder et al., 1974; Groves and Rebec, 1976; Seiden et al., 1993). One well-known type of hallucination induced by AMPH and its derivatives is formication—the sensation of “bugs” biting or crawling on the skin (Ellinwood, 1967; Smith and Crim, 1969; Stanciu et al., 2015). Amphetamine also induces what have been speculated to be hallucinations in non-human animals, including monkeys (Nielsen et al., 1983), rats (Nielsen et al., 1980), and mice (Tadano et al., 1986). Understanding the cellular mechanisms that cause neural networks to generate false perceptions is of great importance to both clinical neuroscience and behavioral biology. Unfortunately, since animals cannot report their subjective experiences, little progress has been made on this topic.

**FIGURE 1:**
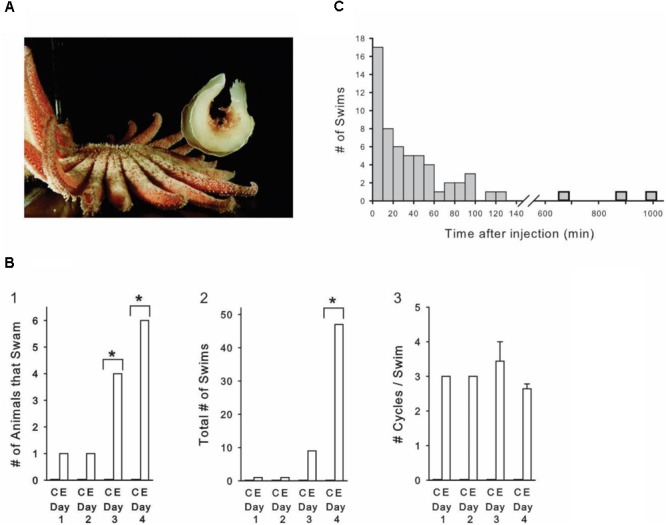
Amphetamine induces sporadic, spontaneous escape swims in freely behaving animals. **(A)** *Tritonia* at maximal dorsal flexion during an escape swim triggered by skin contact with a predator, the seastar *Pycnopodia helianthoides*. **(B)** Progressive AMPH dose experiment. **(B1)** The number of AMPH-injected animals displaying spontaneous swims significantly increased over the course of the 4-day experiment, while controls never swam. **(B2)** The total number of swims in the AMPH-injected group significantly increased over the course of the 4-day experiment. **(B3)** The number of cycles per swim was not affected by the progressive AMPH regimen. AMPH dose on day 1: 5 mg/kg, day 2: 10 mg/kg, day 3: 20 mg/kg, day 4: 40 mg/kg. C, Controls; E, Experimentals. **(C)** Time after AMPH injection of all 58 spontaneous swims that occurred in the progressive dose experiment. AMPH-induced swims occurred sporadically, with the majority occurring more than 20 min after the injection, and nearly one-fourth occurring an hour to many hours after. Asterisks indicate significance at *p* < 0.05.

*Tritonia diomedea* is a marine nudibranch mollusk attractive for neurophysiological studies because of its large pigmented neurons, many of which are individually identifiable from animal to animal. Upon skin contact with its seastar predators, *Tritonia* launches a rhythmic escape swim consisting of a series of alternating ventral and dorsal whole-body flexions (**Figure 1A**). The animal rarely displays this behavior spontaneously. Here we demonstrate that *Tritonia* injected with large or repeated doses of amphetamine (AMPH) launch sporadic escape swims in the absence of any apparent stimulus. The neural circuit mediating this behavior is well understood (**Figure 3A**; Getting, 1983; Frost et al., 2001) and can be studied in deafferented brain preparations where real stimuli can play no role. This allowed us to investigate the neural basis of these unusual drug-induced escape behaviors.

## Materials and Methods

### Behavioral Experiments

*Tritonia diomedea* were obtained from two sources. Those used in the initial pilot behavioral experiment were collected near Dash Point, Puget Sound, WA and maintained in running seawater tanks (11–13°C) at Friday Harbor Laboratories, Friday Harbor, WA, United States. Those used in all remaining experiments were obtained from Living Elements, Vancouver, BC, Canada, and maintained at 11°C in artificial seawater (Instant Ocean, Aquarium Systems, Mentor, OH, United States) at Rosalind Franklin University of Medicine and Science. Animals were injected into the body cavity near the buccal mass with either artificial seawater or D-amphetamine sulfate (Sigma) mixed in artificial seawater. Fresh stock solutions of AMPH were prepared each day. Animals were injected to produce the desired concentration of AMPH (3–40 mg/kg), assuming full diffusion into the animal’s volume, calculated as 1 ml per gm of body weight. Controls were injected with a weight-equivalent amount of artificial seawater. A test animal injected with fast green (Sigma) stained completely, indicating that injected substances do spread from the hemocoel throughout the body tissues. For comparison purposes, common doses of D-amphetamine used in behavioral studies in vertebrate animals range from 1 – 20 mg/kg i.p. (Randrup and Munkvad, 1967), and human amphetamine abusers have been estimated to experience a dosage range of 5–25 mg/kg/day (Trulson and Jacobs, 1979). In mammals, D-amphetamine equilibrates in brain tissue at a higher concentration than the injected i.p. concentration. For example, an 8 mg/kg i.p. injection in rats reaches 25 mg/kg in the brain (Maickel et al., 1969), a value slightly higher than the Day 3 dose of our progressive behavioral experiment.

### Electrophysiological Experiments

The cellular experiments utilized both semi-intact animal and isolated brain preparations. Semi-intact preparation. This consisted of the brain and body, but with the internal organs removed. This preparation was used to obtain intracellular recordings from swim circuit neurons during swim motor programs (SMPs) elicited by natural skin stimulation. The details of this dissection procedure were as previously described (Lee et al., 2012). Isolated brain preparation. The brain, consisting of the fused cerebral–pleural ganglia and the pedal ganglia (with the pedal–pedal commissures cut), was dissected from the animal and pinned dorsal side-up in a Sylgard-lined recording chamber perfused with artificial seawater at 4–6°C. After dissecting away the connective tissue sheath covering the dorsal side of the cerebral–pleural ganglia, a polyethylene suction electrode was attached to left pedal nerve 3 (for nomenclature, see Willows et al., 1973a). The perfusion temperature was then raised to 11°C for the recording session. Intracellular recordings were made with 15–40 MΩ electrodes filled with 3 M KCl or 3 M *K*-acetate. Neurons were identified on the basis of their location, size, color, synaptic connections with other identified neurons, and activity during the SMP, as described previously (Getting, 1983; Frost and Katz, 1996; Frost et al., 2001). Swim motor programs were elicited by applying a 10 Hz, 2 s train of 5 ms 10 V pulses to the suction electrode attached to pedal nerve 3. The AMPH was prepared in artificial seawater at the concentration to be used, and applied via a gravity-driven perfusion system by switching a stopcock between instant ocean and AMPH sources. Data were digitized at 1000 Hz with a Biopac MP150 data acquisition system. Normal saline consisted of (in mM): 420 NaCl, 10 KCl, 10 CaCl_2_, 50 MgCl_2_, 10 HEPES, pH 7.6, and 11 D-glucose. In 0 calcium experiments, the calcium was replaced with the same concentration of either CoCl_2_ or BaCl_2_. Experiments applied AMPH at concentrations ranging from 10 to 1000 μm, similar to the range of 1 to 300 μM used in some vertebrate electrophysiological studies (Mercuri et al., 1989). Throughout results, means are reported ± standard error.

## Results

### AMPH Induces Sporadic, Spontaneous Escape Swims in Freely Behaving *Tritonia*

Skin contact with the tube feet of its seastar predator triggers *Tritonia’s* escape swim, consisting of an alternating series of ventral and dorsal whole-body flexions that propel it away to safety (**Figure 1A**). The escape swim has a high threshold, and in laboratory tanks does not normally occur in the absence of suitably aversive skin stimuli, which include predator contact, bites from conspecifics, or strong salt applied to the skin. We were therefore intrigued to find that *Tritonia* occasionally exhibited spontaneous escape swims in the minutes to hours after being injected with AMPH. In an initial pilot experiment, 25 drug-naïve experimental animals received AMPH injections (3 -15 mg/kg in a saline vehicle), after which they were filmed for 2 h. Some of the animals received additional injections at later times and were again filmed. In response to 48 total injections, 9 of the animals spontaneously swam at least once, with 19 spontaneous swims recorded overall. The swims ranged from 2 – 11 flexion cycles in duration, typical of stimulus-elicited escape swims in this animal. None of the 10 control animals receiving weight-matched injections of the saline vehicle swam.

In a second experiment (**Figures 1B,C**), 10 drug-naïve experimental animals were injected with progressively increasing AMPH doses (see **Figure 1** legend for details), once-per-day for 4 days (mean weight = 95.0 g, range 45 – 200 g). A group of 10 control animals were injected on the same schedule with the saline vehicle (mean weight = 95.0 g, range 15 – 130 g). During the experiment, animals were individually housed in 2 rows of 5 compartments that were pressed against the clear front wall of their home aquarium, where they could be filmed 10 at a time. All animals were filmed continuously with time-lapse video for 4 days (white light 12 h, red light, 12 h), allowing every swim in every animal to be observed over this period. Control and experimental animals were randomly distributed among the different compartments, and the individual viewing the videotapes was blind to which animals received AMPH vs. artificial seawater. All animals were drug-naïve at the start of the experiment.

As in the pilot experiment, several AMPH-injected animals displayed spontaneous swims of 2 or more cycles in the absence of any apparent stimulus, while saline-injected animals never swam. On the first day, 1 of the 10 experimental animals swam after AMPH injection, whereas by day 4, 6 animals swam (**Figure 1B1**). Over the course of the experiment, there was a significant overall difference in the number of animals that swam in the experimental group (*p* < 0.01, Cochran *Q* Test for dichotomous nominal scale data). Day-by-day between-group comparisons indicated that AMPH injections produced a significant increase in the number of animals that swam on day 3 and day 4 (*p* = 0.043 and *p* = 0.005, respectively, Fisher-exact Test, One-tailed). In addition, the total number of swims markedly increased over the course of the experiment (**Figure 1B2**). A two-way repeated measures ANOVA indicated a significant interaction between the AMPH vs. saline injected groups and treatment day [F(3,54) = 4.708, *p* = 0.005]. *Post hoc* Student–Newman–Keuls comparisons between the AMPH and saline groups for each day indicated that the number of SMPs was significantly different on Day 4, the highest dose of AMPH. Thus, while one animal swam once on day 1, by day 4, six animals swam a total of 47 times (*p* < 0.001). In vertebrates, AMPH is well known to produce behavioral sensitization – increased responsiveness over time when the drug is administered in repeated fashion (Robinson and Becker, 1986). We did not attempt to determine whether sensitization contributed to the increased responsiveness observed with our progressive-dose drug regimen. A one-way repeated measures ANOVA indicated that, in spite of the above effect of this progressive AMPH administration regimen on swim occurrence, it had no effect on the number of cycles per swim [*F*(3,8) = 1.349, *p* = 0.325], which averaged 3.0 ± 0.2 across the 4 days of the experiment (**Figure 1B3**).

A notable feature of the AMPH-induced swims was their unpredictability. Rather than occurring immediately after injection, as swims do when *Tritonia* are injected with the neurotransmitter serotonin (McClellan et al., 1994), the AMPH-induced swims occurred sporadically, anywhere from several minutes to several hours following injection of the drug. The time after injection for all 58 AMPH-induced spontaneous swims in the progressive dose experiment is shown in **Figure 1C**. While 17 swims occurred in the first 10 min after injection, the majority occurred later, with 27 occurring more than 30 min following injection, including 3 that occurred at 11.2, 14.7, and 16.5 h post-injection.

### Other AMPH-Induced Behaviors

Amphetamine is well known to produce unusual and repeated stereotyped behaviors in vertebrates, including twitching, rearing, and biting (Groves and Rebec, 1976; Rebec and Bashore, 1984; Seiden et al., 1993). A final behavioral experiment focused on whether AMPH elicits any repetitive stereotyped behaviors in *Tritonia*. Seven drug-naïve experimental animals were injected with a single dose of 20 mg/kg AMPH (mean weight = 86.0 g, range 20–232 g), while 7 controls were injected with artificial seawater (mean weight = 82.3 g, range 22–252 g). After injection, each animal was placed in a Plexiglas box and filmed for 3 h using a tripod-mounted camera and a time lapse VCR to record general activity. In addition, during the first hour an observer visually monitored the animal’s mouth region, using a mirror as needed through the transparent bottom of the tank to record instances of spontaneous mouth opening and/or biting. After all animals were filmed separately, videos of saline- and AMPH-injected pairs were mixed into side-by-side videos to allow simultaneous viewing at 24x speed in order to determine whether there were characteristic effects of AMPH on ongoing behavior.

**FIGURE 2:**
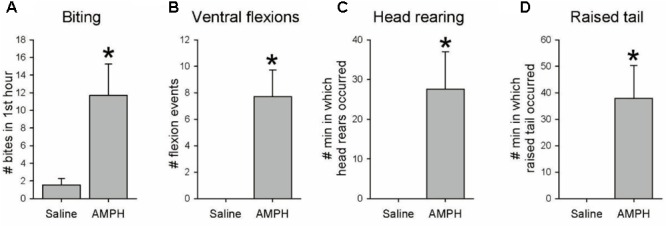
Other behavioral effects of amphetamine. Seven drug-naïve animals were injected with 20 mg/kg AMPH, and seven others with a weight-matched amount of saline vehicle. Animals were videotaped for 3 h after injection, with the mouth area watched live for the first hour. **(A–D)** AMPH-injected animals exhibited significantly more spontaneous biting (*p* = 0.016), non-swimming single ventral flexions/twitches (*p* = 0.002), head rearing (*p* = 0.013) and crawling with raised tail (*p* = 0.010). Asterisks indicate significance at *p* < 0.05.

Amphetamine-injected animals displayed several stereotypic behaviors that were either unique to the drug, or occurred with much greater frequency than in saline-injected controls. *Biting*. During the hour of direct visual observation of their mouth region, AMPH-injected animals exhibited significantly increased spontaneous mouth opening and/or biting vs. controls (**Figure 2A**; mean = 11.7 ± 3.5 vs. 1.6 ± 0.7 events, *t*-test, *t* = 2.81, *p* = 0.016), involving mouth opening events which often included the full odontophore grasping and radular scraping components of a normal bite, but with nothing in contact with the mouth region. During the full 3 h of post-injection videotaped behavior AMPH-injected animals exhibited several additional behaviors not normally seen. *Ventral flexions*. Drug-injected animals displayed several spontaneous single ventral twitches or flexions. (**Figure 2B**; mean = 7.7 ± 2.0 vs. 0.0 ± 0.0 flexions, *t* = 3.86, *p* = 0.002). *Head rearing*. AMPH-injected animals often crawled with their front foot margin and oral veil raised above the substrate, which we referred to as head rearing behavior. To document this, we counted the number of minutes when any instance of head rearing behavior occurred during the 3-h post-injection observation period. AMPH injected animals showed significantly more head rearing than controls (**Figure 2C**; mean = 27.6 ± 9.4 vs. 0.0 ± 0.0 min in which rearing events occurred, *t* = 2.92, *p* = 0.013). *Raised tail*. AMPH-injected animals also often crawled with their tail raised off the substrate, a behavior not seen in the saline-injected controls (**Figure 2D**; mean = 37.9 ± 12.5 vs. 0.0 ± 0.0 min in which such events occurred, *t* = 3.03, *p* = 0.010). These results appear consistent with amphetamine’s ability to induce repeated stereotyped behaviors in vertebrates, in spite of *Tritonia*’s markedly different invertebrate CNS organization.

### The AMPH-Induced Swim Initiates From Within the Brain, Rather Than in Response to a Real Skin Stimulus

**FIGURE 3:**
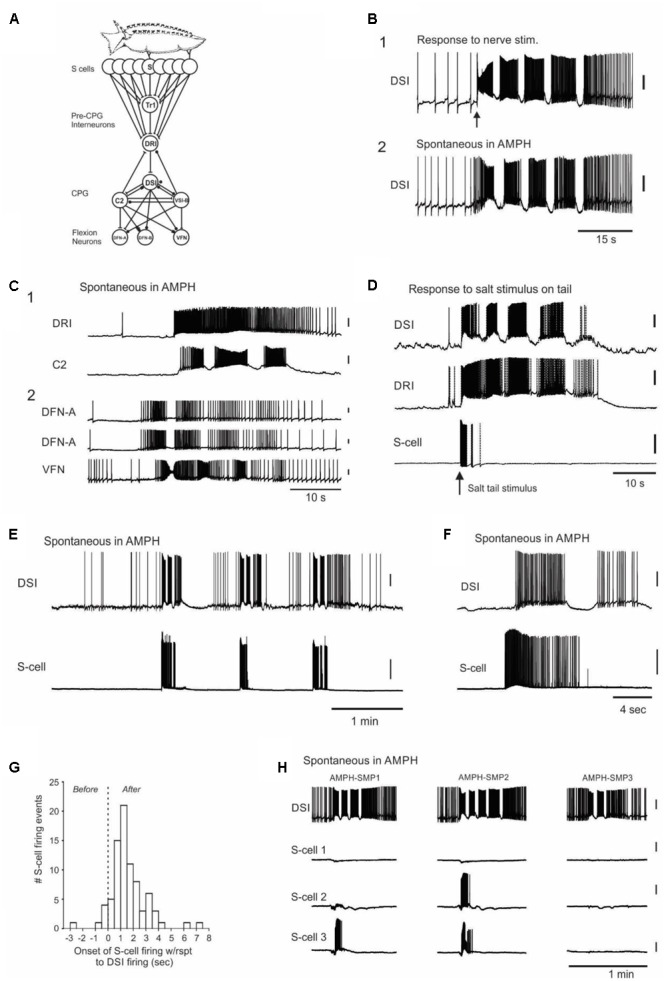
Neurophysiological evidence that the AMPH-induced swims originate within the CNS, with spontaneous bursts in the normally silent afferent neuron population that detects the animal’s seastar predators. **(A)** *Tritonia* escape swim circuit. Skin stimuli elicit the motor program by exciting the S-cells (afferent neurons) in the brain, which in turn activate pre-CPG command interneurons, CPG interneurons and efferent flexion neurons. S, S-cells; Tr1, Trigger-type 1 command neuron; DRI, dorsal ramp command neuron; DSI, dorsal swim interneuron; C2, Cerebral neuron 2; VSI-B, Ventral swim interneuron type B; DFN-A, Dorsal flexion neuron type A; DFN-B, Dorsal flexion neuron type B; VFN, Ventral flexion neuron. **(B)** Similarity of sensory-elicited vs. AMPH-induced swim motor programs (AMPH-SMPs). **(B1)** Stimulus-elicited SMP in normal saline, elicited via brief suction electrode stimulation (10 Hz, 1 s, 10 V) of Pedal Nerve 3, a peripheral nerve containing S-cell axons. **(B2)** Spontaneous AMPH-SMP that occurred 50 min after switching perfusion from normal saline to 50 μM AMPH saline. The two recordings are from different preparations. As can be seen here and in other panels, spontaneous AMPH-SMPs are very similar in appearance to normal, stimulus-elicited SMPs. **(C)** A survey of circuit neurons traced the origin of the AMPH-SMP to the CPG or more afferent network loci. **(C1)** Simultaneous recording from the pre-CPG command neuron DRI and the CPG interneuron DSI during a spontaneous AMPH-SMP. **(C2)** Simultaneous recording in a different preparation from three flexion neurons during a spontaneous AMPH-SMP. Both experiments in 1 mM AMPH. **(D)** Swim motor program elicited by salt applied to the skin in a semi-intact animal preparation. Stimulus-elicited SMPs begin with a burst of action potentials in the S-cells, which converge onto the single command neuron DRI that in turn directly drives the DSI neurons of the CPG. **(E)** Three consecutive spontaneous motor programs in an isolated brain preparation recorded in 50 μM AMPH. Each AMPH-SMP began with a burst of spikes in the recorded S-cell. **(F)** Recording of a spontaneous S-cell burst that began shortly before the onset of the AMPH-SMP. **(G)** The time of onset of all recorded S-cell bursts with respect to the first action potential of the speed-up of DSI firing rate that signaled AMPH-SMP onset. **(H)** Simultaneous recording from DSI and three S-cells during three spontaneous AMPH-SMPs in an isolated brain perfused with 100 μM AMPH. Different combinations of S-cells initiate each AMPH-SMP, consistent with a shifting body location for the perceived but non-existent predator contact. The motor programs occurred at 7.0, 10.9, and 30.1 min after the onset of AMPH perfusion. All but **(D)** are from isolated brain preparations. All vertical scale bars = 20 mV.

While the AMPH-induced swims in the behavioral experiments appeared to be spontaneous in origin, it was possible that the drug enhanced the animals’ awareness of, or responsiveness to, real skin irritants that are normally below threshold for eliciting the swim. This issue has plagued interpretation of the origin of “spontaneous” AMPH-induced behaviors in vertebrate studies (see Discussion). To address this issue we next tested whether AMPH would induce SMPs in deafferented, isolated brain preparations, where sensations elicited by skin stimuli cannot occur. SMPs elicited by nerve stimulation in the isolated brain preparation (**Figure 3B1**) were similar in appearance to those elicited by aversive skin stimuli in semi-intact animal preparations (**Figure 3D**), consistent with the well-documented negligible role of sensory feedback in this centrally generated motor program (Dorsett et al., 1973; Frost et al., 2001).

In decades of work with *Tritonia* isolated brain preparations, we had never observed a spontaneous SMP in normal saline. It was therefore striking that in all 30 drug-naive isolated brains in which it was attempted, perfusion with 50 μM to 1 mM AMPH led to several (range = 1–19 per preparation) spontaneous, AMPH-induced swim motor programs (AMPH-SMPs) (**Figure 3B2**; 50 μM: 7.0 ± 1.6 AMPH-SMPs per preparation, 2.9 ± 0.2 cycles per motor program, range = 2–4 cycles, 5 preparations; 100 μM: 10.4 ± 1.8 AMPH-SMPs per preparation, 3.0 ± 0.5 cycles per motor program, range = 2–7 cycles, 7 preparations; 1 mM: 3.3 ± 0.7 AMPH-SMPs per preparation, 3.1 ± 0.2 cycles per motor program, range 2–7 cycles, 18 preparations). In these experiments, each brain was exposed once to a single concentration of AMPH. AMPH perfused at 10 or 20 μM did not induce AMPH-SMPs in single experiments tried at each of these lower concentrations, but more work is needed to reliably determine the threshold dose. AMPH-SMPs were similar in appearance to SMPs elicited by real sensory input – they began abruptly from a normal baseline of neuronal activity and then proceeded through several cycles of rhythmic firing (**Figure 3B**). From these isolated brain results we conclude that the drug-induced swims observed in AMPH-injected intact behaving animals appear to be triggered, not by actual skin stimuli, but instead by spontaneous activity originating within the nervous system.

### The AMPH-Induced Swim Motor Program Originates With Spontaneous Bursts of Activity in the Swim Afferent Neurons

We next sought the site of origin of the AMPH-SMPs in the swim circuit. Because direct intracellular stimulation of several individual command and CPG interneurons can effectively bypass the S-cells and elicit the SMP in normal saline (Getting, 1977; Frost et al., 2001; Katz et al., 2004), there were multiple potential sites of origin of the AMPH-SMP in the swim circuit. We therefore obtained intracellular recordings from neurons at all hierarchical levels of the swim circuit during spontaneous AMPH-SMPs to determine where the circuit activity originated. For example, if the AMPH-SMP originates in the CPG, then the upstream neurons that normally fire to trigger the skin-elicited SMP would be expected to remain largely silent. Over the course of 38 preparations, which included those described above, we obtained multiple intracellular recordings from most of the known members of the swim circuit during spontaneous AMPH-SMPs, including the DRI swim command neurons (*N* = 2), the C2 (*N* = 9) and DSI (*N* = 44) CPG neurons, and the DFN (*N* = 11) and VFN (*N* = 14) flexion neurons. We found that all sampled interneurons and flexion neurons participated during the spontaneous AMPH-SMP (**Figures 3B,C**) as they normally do in response to real sensory input (**Figures 3B,D**; Getting, 1983; Frost et al., 2001).

Having traced the origin of the spontaneous AMPH-SMP as far as the swim command neurons, we next turned to their input, the well-characterized swim afferent neurons (S-cells). The S-cells have their cell bodies located in a cluster on the dorsal side of each pleural ganglion (Getting, 1976; Megalou et al., 2009). Each pseudounipolar S-cell sends one or more axons out peripheral nerves to innervate specific regions of the skin (Getting, 1976; Frost et al., 2003). Within the brain, each S-cell makes monosynaptic excitatory connections onto the Tr1 and DRI command neurons to initiate the SMP (**Figure 3A**) (Frost and Katz, 1996; Frost et al., 2001). **Figure 3D** shows how S-cells, together with the swim command neuron DRI and CPG neuron DSI fire in response to an SMP-initiating aversive salt stimulus applied to the skin in a semi-intact animal preparation. Such stimuli elicit a vigorous burst of firing in those S-cells having receptive fields in the stimulated skin region. The S-cell burst then terminates soon after the SMP gets underway.

In 18 of the 38 isolated brain preparations comprising the above swim network survey, one to three S-cells were simultaneously recorded together with a DSI neuron, which was included to indicate the onset time of each AMPH-SMP, as well as its number of cycles. Across these preparations, AMPH perfusion induced a total of 114 spontaneous SMPs of 2 or more cycles (mean = 8.8 SMPs ± 1.9, range = 1–19 per preparation), yielding a dataset of 238 recordings of how 40 S-cells did or did not fire during AMPH-SMPs. In total we recorded 81 S-cell firing events during 59 AMPH-SMPs in 18 preparations. These firing events were typically vigorous (mean = 69.9 ± 5.2 spikes; range = 3–223 spikes), with firing rates reaching 33 Hz.

S-cells in the isolated brain are silent at rest, and their only known synaptic input is inhibitory, from the normally silent Pl9 neuron that gets its input from the S-cells (Frost et al., 2003). We therefore did not expect them to fire in association with the AMPH-SMP, when there is no possibility of skin stimulation. However, of the above 40 S-cells, 12 (30%) fired a burst of spikes before or during the initial part of the first spontaneous AMPH-SMP that occurred during their recording (**Figures 3E–H**). Assuming our sampling was random from the 80 S-cells estimated to be in the recorded pleural ganglion (Getting, 1976), this suggests that approximately 24 S-cells erupt into activity at the time of onset of the AMPH-SMP. Since a prior study found that directly driving a minimum of 5 S-cells is needed to initiate the SMP in normal saline (Getting, 1976), this large-scale firing event in the S-cell population appears more than sufficient to trigger the AMPH-SMP.

In isolated brain studies of the swim network in normal saline, the motor program is typically triggered by trains of short electrical pulses applied to PdN3, such as in **Figure 3B1**. In that highly artificial case, 100% of the directly driven S-cells will start firing before the CPG’s DSI neurons, since the latter are two synapses downstream from the S-cells (**Figure 3A**). **Figure 3G** shows the time of onset of all 81 recorded S-cell bursts with respect to the first action potential of the speed-up of DSI firing rate that signals the first hint of motor program onset. This S-cell firing, because it was not forced by direct stimulation of S-cell axons in a peripheral nerve, was not synchronous in onset. The very first S-cells to fire, such as those first responders shown in **Figure 3G**, would act to start increasing the DSI firing rate. Then, as increased numbers of S-cells rapidly join the population burst, they drive the accelerating DSI activity that becomes the first motor program cycle.

The S-cells somatotopically innervate the body surface (Getting, 1976; Slawsky, 1979; Frost et al., 2003), thus each cell normally informs the animal of a stimulus at a specific region of the body. Of the 11 S-cells that fired in preparations with multiple AMPH-SMPs, 9 did so in some SMPs but not others (**Figure 3H**). From this we conclude that the body location of the perceived skin stimulus apparently shifts from episode to episode (see Discussion).

In a further test of the hypothesis that spontaneous bursts in the S-cells are the origin of the AMPH-SMP, we also examined whether AMPH could induce the motor program when applied only to a small well beside the isolated brain containing the cut end of PdN3, which remained attached to the brain via a Vaseline-sealed slit. Many S-cells send their peripheral axons in PdN3 to innervate the skin. In the 2 preparations in which this was tried, exposing the nerve alone to 1 mM AMPH led to 14 total spontaneous SMPs, during which 3 of 6 recorded S-cells fired a burst at swim onset. As a peripheral nerve, PdN3 primarily contains the axons of afferent and efferent neurons, so this observation supports our conclusion that the AMPH-SMP originates with spontaneous bursts in the S-cells, rather than with network interneurons, whose processes are not known to travel in peripheral nerves.

### Possible Mechanisms Contributing to the AMPH-Induced Afferent Neuron Population Burst

Prior studies estimated the size of the S-cell population to be approximately 160 neurons (∼80 per pleural ganglion) (Getting, 1976, 1983). The above finding that 30% of recorded S-cells fired a burst of spikes before or during the initial part of at least one AMPH-SMP suggests that a sizable portion of the S-cell population spontaneously erupts into activity from a silent baseline to trigger each AMPH-SMP. AMPH thus transforms the normally silent and non-interactive S-cell population into one that is sporadically eruptive. Further experiments exposed possible contributing processes.

In 15 experiments from the above dataset, S-cells were impaled and driven at regular intervals with 3 or 5 s depolarizing constant current pulses (36 total S-cells, 2 – 3 per preparation) while recording a DSI CPG neuron to monitor SMP occurrence during AMPH perfusion. Depending on the preparation, current pulses were administered at either 1, 2, or 5 min intervals, beginning several minutes before, and continuing for several minutes after the start of perfusion of either 0.05 mM (*N* = 5 preparations) or 1 mM (*N* = 10 preparations) AMPH.

#### Enhanced S-Cell Efficacy and Excitability

**FIGURE 4:**
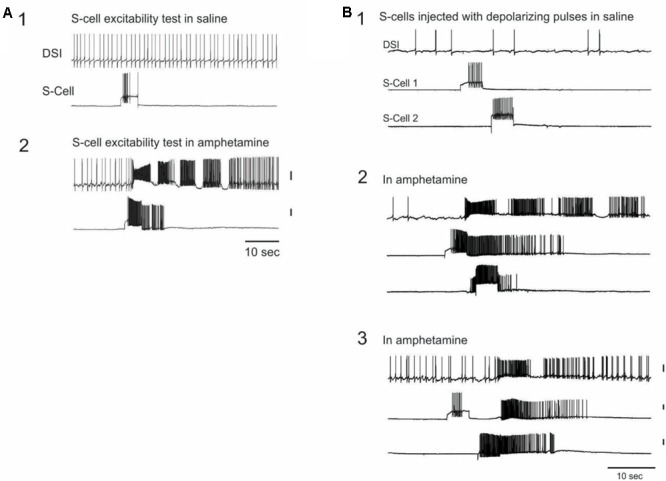
Single S-cells can trigger the AMPH-SMP. **(A)** In AMPH, S-cells respond to constant current injections with increased firing, which can continue after the current injection and can trigger the swim motor program. The same two neurons are simultaneously recorded across both panels. **(B)** In AMPH, S-cells can excite strong firing in other S-cells. The same three neurons were simultaneously recorded across the panels. **(B1)** in saline, both S-cells responded to the current injection with modest firing. **(B2)** in 1 mM AMPH, the firing of the first neuron to the constant current test pulse triggered an SMP. The firing continued after the test pulse and also spread to the other S-cell. **(B3)** Injecting the same constant current pulses triggered a one-cycle SMP that originated this time from the other S-cell. The amount of injected current was constant across panels for each neuron. All vertical scale bars = 20 mV.

A striking finding was that in AMPH, 14 of the 36 S-cells (38.9%) across 10 preparations triggered an SMP in response to the firing of that single neuron driven by a current pulse (**Figure 4A**). Five of these 14 S-cells, all in different preparations, triggered SMPs on multiple trials (range 2–4 trials). Because SMPs also occurred spontaneously in the presence of AMPH (mean = 4.73 ± 0.76 SMPs, range = 1–10 per preparation), an SMP was considered to have been triggered by the S-cell current pulse if the speed-up of DSI tonic firing signaling motor program onset began within 2 s after the end of the S-cell current injection. Such triggering of SMPs by single S-cells has never been observed by us in normal saline, either during this study, or across several years of work with S-cells (Frost et al., 2001, 2003; Megalou et al., 2009; Lee et al., 2012). Ten of the 36 recorded S-cells, in 7 preparations, also exhibited firing that continued beyond the end of the current pulse in AMPH (mean = 24.08 ± 8.55 extra spikes, range = 1–101), a phenomenon also never observed in normal saline in these experiments or in prior work (**Figures 4A,B**).

Occasionally the S-cell firing elicited by the constant current pulses appeared to spread to other recorded S-cells in AMPH (**Figure 4B**). This occurred with 5 (13.9%) of the 36 stimulated S-cells on 1 or more current pulse trials, in 3 of the 15 preparations, involving both 0.05 and 1.0 mM AMPH. The mechanism of this rapid spread of firing in the S-cell population remains unknown. Prior studies have reported no direct excitatory synaptic connections among the S-cells (Getting, 1976), consistent with our own observations before this study.

#### AMPH-Dependent Plateau Potentials in S-Cells

**FIGURE 5:**
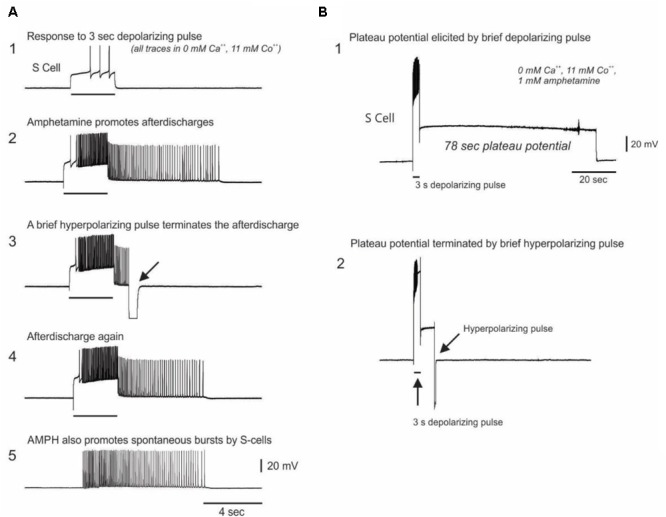
Evidence for AMPH-induced plateau potential properties in S-cells. **(A)** Top trace: In saline in which calcium was replaced with the calcium channel blocker cobalt, constant current test pulses elicited modest firing. Second trace: In 0 Ca^2+^, 11 mM Co^2+^, 1 mM AMPH, the same test pulse elicited greater firing, followed by post-burst firing that long outlasted the test pulse. Third trace: the prolonged post-burst firing could be abruptly terminated by a brief hyperpolarizing pulse, a characteristic feature of plateau potentials. Fourth trace: Another test pulse again elicited post-burst firing. Fifth trace: 32% of the S-cells recorded in 0 calcium AMPH emitted sporadic spontaneous bursts. All traces are from the same neuron in one preparation. **(B)** In 0 calcium AMPH, injecting sufficient depolarizing current to fully accommodate the action potential often triggered a pronounced plateau potential which could last over a minute. This plateau potential could be terminated early with a brief injection of hyperpolarizing current. Both traces are from the same neuron.

As a further test of whether AMPH acts directly on the S-cells, we repeated the prior constant current test pulse protocol in calcium-free AMPH saline, in which the normal 11 mM calcium chloride was replaced by either 11 mM cobalt chloride or 11 mM barium chloride. Twenty five S-cells were recorded in 0 calcium saline in 8 new preparations (range = 1–4 S-cells per preparation). Before the addition of 1 mM AMPH, 3–5 s depolarizing constant current pulses delivered at 1–2 min intervals elicited S-cell firing that always ceased with the end of the pulse (**Figure 5A1**). After 1 mM AMPH was added, 12 of the 25 S-cells sporadically exhibited firing that continued beyond the end of the current pulse (mean of the largest such event for each cell = 30.92 ± 8.29 extra spikes, range 1–84; **Figure 5A2**).

The sporadic nature of this post-current injection firing in AMPH may be associated with AMPH-induced plateau potential properties in the S-cells that are variably triggered by the current pulses. Plateau potentials are induced in many invertebrate and vertebrate neurons by monoamine and other modulatory transmitters (Kiehn, 1991), and cause cells to exhibit sustained firing in response to brief inputs. Once triggered, plateau potentials either spontaneously terminate, or can be terminated by brief hyperpolarizing inputs. In calcium free, 1 mM AMPH saline the prolonged S-cell firing that continued after injections of depolarizing current could be abruptly terminated by injecting brief hyperpolarizing current pulses (**Figures 5A2–A4**), suggesting they are plateau potential based. In 9 of the above 25 S-cells examined in 0 calcium AMPH saline, injecting sufficient depolarizing current to fully accommodate the S-cell action potential sporadically evoked large-amplitude spike-free plateau potentials that outlasted the current pulse by several seconds to over 1 min (**Figure 5B1**). In 5 out of 6 S-cells in which it was attempted, these large depolarization-induced plateau potentials were abruptly terminated by brief hyperpolarizing current pulses (**Figure 5B2**, 5 preparations). While many plateau potentials are calcium-dependent (Kiehn, 1991), calcium-independent examples, such as shown here, have also been described in both vertebrates (Llinas and Sugimori, 1980; Hoehn et al., 1993) and invertebrates (Angstadt and Choo, 1996).

Consistent with AMPH inducing instability in the S-cell population through direct action on these cells, when isolated from spike-mediated chemical synaptic inputs by 0 calcium saline S-cells were still observed to sporadically burst spontaneously. Eight of the 25 S-cells examined in 0 calcium AMPH gave forth spontaneous bursts (mean of the largest such burst for all such cells = 36.00 ± 9.91 spikes in a burst duration of 4.37 ± 1.06 s; range = 9–84 spikes; mean max frequency = 16.05 Hz, **Figure 5A5**). Spontaneous bursts were never observed during the pre-AMPH testing period in 0 calcium saline.

Taken together, the above 0 Ca^2+^ results are consistent with the hypothesis that AMPH exerts its effects either directly on the S-cells, or perhaps on as-yet unidentified monoaminergic terminals synapsing directly onto the S-cells. In both invertebrates and vertebrates, amphetamine acts as an indirect monoaminergic agonist, promoting calcium-independent transmitter release from the presynaptic terminal, in part by reversing presynaptic monoaminergic reuptake transporters (Seiden et al., 1993; Sulzer et al., 1995). Both serotonin and dopamine are present in the *Tritonia* CNS (McCaman et al., 1973; Sudlow et al., 1998; Fickbohm et al., 2001). Dopamine has been implicated in the animal’s cilia-mediated crawling behavior (Woodward and Willows, 2006), but was found to inhibit the nerve-elicited SMP in isolated brain preparations (McClellan et al., 1994). Serotonin’s role in *Tritonia’s* escape swim has been well-studied. Serotonin elicits the animal’s escape swim (McClellan et al., 1994), and the serotonergic DSI neurons of the swim CPG can drive the escape SMP and produce intrinsic neuromodulation of other neurons in the swim circuit (Katz et al., 1994; Katz, 1998). Possible direct modulation of the S-cells by either of these monoamine transmitters has not yet been examined.

## Discussion

### Evidence for an Invertebrate Model of Drug-Induced Hallucinations

The present study originated from curiosity about how AMPH, a commonly abused drug in humans, would act in a well-studied invertebrate with a highly tractable nervous system. We found that AMPH induced spontaneous escape swims in freely behaving animals, in the absence of any apparent stimulus. More surprisingly, we found that drug-induced escape SMPs sporadically occurred in deafferented isolated brain preparations, and traced their origin to spontaneous bursts in the afferent neuron population that normally informs the animal of skin contact with its seastar predators. Here we present an argument that *Tritonia’s* AMPH-induced spontaneous swims are initiated in response to drug-induced perceptions of non-existent aversive skin stimuli, i.e., hallucinations.

Hallucinations were first formally described as perceptions of stimuli that do not actually exist (Esquirol, 1965). The DSM-IV definition is “a sensory perception that has the compelling sense of reality of a true perception, but that occurs without external stimulation of the relevant sensory organ” (DSM-IV, 2000). We suggest that *Tritonia’s* response to AMPH conforms to both components of this definition. Since the origin of the AMPH-SMPs was traced to spontaneous S-cell bursts in isolated brain preparations, they involve sensory neuron activity in the absence of an actual stimulus. Moreover, because the animal responds by launching its high-threshold escape swim, this sensory activity is clearly both perceived by and compelling to the animal. Since S-cells respond most strongly to skin contact with a chemical substance in the tube feet of the animal’s seastar predators (**Figure 3B**; Getting, 1976), *Tritonia‘s* AMPH-induced hallucinations appear to be of predator contact. To our knowledge, the present study represents the first evidence for hallucinations in an invertebrate.

Many stimulus-elicited behavioral responses can be classed as simple, graded reflexes, in which response magnitude is proportional to stimulus strength. In contrast, *Tritonia’s* escape swim is a complex, high threshold, all-or-none command neuron driven behavior (Frost and Katz, 1996; Frost et al., 2001) that only occurs to suitably aversive stimuli. For example, *Tritonia* do not swim in response to either tactile (Willows et al., 1973b; Mongeluzi et al., 1998) or even many tissue damaging skin stimuli (unpublished observations), presumably because of the behavior’s high cost for the animal. Its thrashing, dorsal-ventral body flexions typically lift the animal into water currents that can carry it far away from food and potential mates (Willows, 2001; Wyeth and Willows, 2006). Below threshold stimuli produce graded, reflex withdrawal of the affected body region. Slightly stronger stimuli may elicit bilateral withdrawal of the gills and rhinophores, and even whole-body stiffening, all normal preparatory components of the swim itself, and yet the swim will not be launched unless the stimulus is sufficiently strong or prolonged (Willows et al., 1973b). Taken together, these findings are consistent with the AMPH-elicited swims being launched in response to compelling perceptions of skin contact with non-existent predators.

Behavioral studies of chronic AMPH exposure in vertebrate animals have led investigators to suggest the possible occurrence of AMPH-induced hallucinations in monkeys (Nielsen et al., 1983; Castner and Goldman-Rakic, 1999), cats (Trulson and Jacobs, 1979), rats (Nielsen et al., 1980), and mice (Tadano et al., 1986) (several studies are reviewed in Ellison, 1991). However, such studies have so far been unable to determine whether drug-induced behaviors such as repeated digging at the skin, or turning to stare or vocalize at objects unseen by human observers, represent true perceptual hallucinations, altered perceptions of real stimuli, or motor automatisms. If, in our *Tritonia* studies, AMPH had induced the motor program by activating the swim CPG directly, without prior activation of the sensory neurons, these behaviors would have been classified as automatisms rather than hallucinations. We similarly would not have posited hallucinations had we recorded just one or two spontaneous S-cell action potentials, with no observable effect on downstream circuitry or behavior. Such modest afferent neuron activity is well below threshold for triggering the escape swim behavior (Getting, 1976), and thus could not reasonably be classified as being compelling to the animal.

The majority of our cellular studies of AMPH-induced SMPs involved drug-naïve preparations. AMPH-induced hallucinations in humans, as well as those posited to occur in vertebrate animal studies, characteristically occur after repeated or continuous drug administration (Ellison, 1991). However, several publications, involving both emergency room admissions (Connell, 1958; Gold and Bowers, 1978) as well as controlled drug administration studies in hospital settings (Angrist and Gershon, 1970; Bell, 1973; Snyder et al., 1974; Seiden et al., 1993), have documented the occurrence of hallucinations in response to initial acute exposure to AMPH, at times in individuals believed to have no prior experience with the drug.

Chronic amphetamine abuse can produce delusional parasitosis in humans, involving formication: vivid tactile hallucinations of invertebrates biting or crawling on the skin (Stanciu et al., 2015). Given the encoding function of *Tritonia’s* S-cells, the present study suggests that amphetamines can apparently induce surprisingly similar aversive perceptions, albeit operating at an unconscious level, in invertebrates themselves.

### False Perceptions Need Not Be Conscious to Be Considered Hallucinations

Being an invertebrate, *Tritonia’s* hallucinations are presumably non-conscious. [However, it seems worth noting that the impressive cognitive abilities of certain invertebrates have led many authors to suggest that such organisms may be capable of some degree of conscious awareness (Walters et al., 1981; Griffin and Speck, 2004; Edelman et al., 2005; van Swinderen, 2005; Smith, 2009)]. While the notion of unconscious hallucinations may be unfamiliar, it is well known that sensory information in humans is routed to, and perceived in detail, in both conscious and unconscious brain regions (Sahraie et al., 1997; Anders et al., 2004; Augusto, 2010; Fahrenfort et al., 2017). A well-studied example is that of “blindsight,” in which individuals unable to see due to damage to their primary visual cortex are nonetheless able to use unconscious perception to navigate around obstacles, point to the locations of objects (Cowey, 2010), and even identify the emotional tone of pictures of human faces (Danckert and Rossetti, 2005). Additional studies have demonstrated unconscious visual and tactual perceptual abilities in healthy individuals (Imanaka et al., 2002). Many studies have concluded that human unconscious perception is as richly detailed as conscious perception, able to support perceptual, evaluative and motivational guidance to behavioral choice (Marcel, 1983; Bargh and Morsella, 2008; Hassin, 2013) and even a degree of rational deliberation (Garrison and Handley, 2017) and the setting and pursuit of goals (Custers and Aarts, 2010). In addition to such parallel pathways for processing perception, it is well established that learning and memory have distinct conscious (explicit) and unconscious (implicit) components that are processed, stored and accessed separately (Schacter, 1992; Squire, 2009). In fact, it is often suggested that the unconscious, rather than the conscious mind is actually better suited for reaching decisions involving complex issues – the familiar example of “sleeping on it” to achieve clarity with regard to selecting a best course of action (Dijksterhuis and Nordgren, 2006).

Given that a portion of perception in humans appears to be mediated by unconscious networks, drug-induced spontaneous activity in such networks may reasonably be hypothesized to trigger hallucinations that, while not perceived consciously, might nonetheless affect an individual’s affect and behavior. This notion is consistent with the large psychoanalytic literature on the significant role that “phantasies,” defined as “the primary content of unconscious mental processes,” play in human experience (Spillius, 2001; Ogden, 2011). From this perspective, it seems reasonable to posit that the nervous systems of animals living entirely unconscious lives may also, under the influence of psychostimulants such as AMPH, generate and respond to hallucinations of non-existent stimuli.

### Generalizability of Invertebrate Mechanisms to Higher Animals

Invertebrates have long been successfully used to pursue general principles of nervous system function (Clarac and Pearlstein, 2007). For example, results from marine mollusks have been found to generalize to vertebrates across several levels of complexity, including mechanisms of action potential generation (Catterall et al., 2012), learning and memory (Pittenger and Kandel, 2003; Glanzman, 2010), and even sleep (Michel and Lyons, 2014). *Tritonia* research has identified the first cellular mechanisms mediating prepulse inhibition, an important sensory gating process common to both vertebrates and invertebrates (Mongeluzi et al., 1998; Frost et al., 2003; Lee et al., 2012). Prepulse inhibition deficits are a core feature of schizophrenia, and have been linked to several cognitive disorders of the disease, including psychosis (Braff et al., 2001; Quednow et al., 2008; Ziermans et al., 2011, 2012).

How likely is it that hallucinations will share at least some features in common between invertebrates and humans? One relevant perspective has been raised by several authors—that some brain mechanisms operating in the non-conscious, more ancestral regions of the human brain appear to have changed little through evolutionary time (Reber, 1992; Augusto, 2010). Another is that while the subjective content of human hallucinations varies with their location of origin in the brain, the triggering mechanisms may be more parsimonious. This hypothesis is supported by several decades of electrical microstimulation of cortex during brain surgery in awake humans, which has shown that very different subjective experiences and memories can be elicited by this uniform type of stimulation, simply by varying the locus of stimulation (Curot et al., 2017). Thus, while our example involves AMPH-induced instability in a sensory neuron population, locating the same mechanism in interneuronal networks in different regions of the mammalian brain would be expected to trigger, once elaborated by local cortical processing, conscious hallucinations of diverse and complex character.

Several features of the results seem consistent with the potential for using *Tritonia* to investigate the poorly understood network instability mechanisms that trigger hallucinations. First, the AMPH-induced swims in intact animals, and the corresponding AMPH-induced motor programs in isolated brains occur sporadically and without warning in the minutes to hours after AMPH administration, much as hallucinations of varied causes do in humans. Second, while AMPH can produce elevated excitability in vertebrate neurons (Jahromi et al., 1991; Ma et al., 2013), its effect on *Tritonia’s* S-cells is of particular interest due to its sporadic nature. Even when tested in 0 Ca^2+^ saline, where spike-mediated synaptic inputs can play no role, *Tritonia’s* AMPH-induced S-cell plateau potentials occurred on some test depolarizations and not on others. This trial-to-trial variability of AMPH’s effect on S-cell excitability resembles the sporadic nature of hallucinations themselves. Finally, AMPH is well known in vertebrates to elevate monoamine release, including serotonin (Hernandez et al., 1987; Jones and Kauer, 1999), by reversing transmitter reuptake transporters (Fleckenstein et al., 2007). While we have not determined whether AMPH promotes serotonin release via this mechanism in *Tritonia*, it has been shown to do so with respect to dopamine in the gastropod *Planorbis*, thus this basic mode of action is common to invertebrates (Sulzer et al., 1995). The facts that serotonin injections trigger *Tritonia’s* escape swim, and that serotonin receptors mediate the actions of many classical hallucinogens in vertebrates (Halberstadt, 2015), are consistent with a possible role for this transmitter in mediating AMPH-induced hallucinations in this invertebrate model system.

## Author Contributions

AL conducted the experiments, analyzed the data, and participated in writing the paper. CB conceived of the project, conducted the experiments, and analyzed the data. JW conducted the experiments. WF conducted the experiments, analyzed the data, participated in writing the paper, and made the figures.

## Conflict of Interest Statement

The authors declare that the research was conducted in the absence of any commercial or financial relationships that could be construed as a potential conflict of interest.

## Footnotes

1 https://www.frontiersin.org/research-topics/5495/invertebrate-models-of-natural-and-drug-sensitive-reward
